# Back Pain Consortium (BACPAC): Protocol and Pilot Study Results for a Randomized Comparative-Effectiveness Trial of Antidepressants, Fear Avoidance Rehabilitation, or the Combination for Chronic Low Back Pain and Comorbid High Negative Affect

**DOI:** 10.1093/pm/pnad006

**Published:** 2023-01-30

**Authors:** Ajay D Wasan, Robert R Edwards, Kevin L Kraemer, Jong Jeong, Megan Kenney, Kevin Luong, Marise C Cornelius, Caitlin Mickles, Bhagya Dharmaraj, Essa Sharif, Anita Stoltenberg, Trent Emerick, Jordan F Karp, Matt J Bair, Steven Z George, William M Hooten

**Affiliations:** Department of Anesthesiology and Perioperative Medicine, University of Pittsburgh School of Medicine, Pittsburgh, PA 15206, United States; Department of Anesthesiology, Perioperative, and Pain Medicine, Brigham and Women’s Hospital, Boston, MA 02467, United States; Department of Medicine, University of Pittsburgh School of Medicine, Pittsburgh, PA 15261, United States; Department of Biostatistics, University of Pittsburgh School of Public Health, Pittsburgh, PA 15261, United States; Department of Occupational Therapy, School of Health and Rehabilitation Sciences, University of Pittsburgh, Pittsburgh, PA 15261, United States; Department of Anesthesiology and Perioperative Medicine, University of Pittsburgh School of Medicine, Pittsburgh, PA 15206, United States; Department of Anesthesiology, Perioperative, and Pain Medicine, Brigham and Women’s Hospital, Boston, MA 02467, United States; Department of Anesthesiology and Perioperative Medicine, University of Pittsburgh School of Medicine, Pittsburgh, PA 15206, United States; Department of Anesthesiology and Perioperative Medicine, University of Pittsburgh School of Medicine, Pittsburgh, PA 15206, United States; Department of Anesthesiology, Mayo Medical School, Rochester, MA 55905, United States; Department of Anesthesiology, Mayo Medical School, Rochester, MA 55905, United States; Department of Anesthesiology and Perioperative Medicine, University of Pittsburgh School of Medicine, Pittsburgh, PA 15206, United States; Department of Psychiatry, University of Arizona College of Medicine, Tucson, AZ 85007, United States; Center for Health Information and Communication (CHIC), Health Services Research & Development (HSRD), Richard L Roudebush Veterans Affairs Medical Center, Indianapolis, IN 46202, United States; Department of Medicine, Indiana University School of Medicine, Indianapolis, IN 46202, United States; Department of Orthopaedics, Duke University, Durham, NC 27710, United States; Duke Clinical Research Institute, Duke University, Durham, NC 27701, United States; Department of Anesthesiology, Mayo Medical School, Rochester, MA 55905, United States

**Keywords:** chronic low back pain, negative affect, comparative effectiveness, randomized trial

## Abstract

**Objective:**

Patients with chronic low back pain (CLBP) and comorbid depression or anxiety disorders are highly prevalent. Negative affect (NA) refers to a combination of negative thoughts, emotions, and behaviors. Patients with CLBP with high NA have greater pain, worse treatment outcomes, and greater prescription opioid misuse. We present the protocol for SYNNAPTIC (SYNergizing Negative Affect & Pain Treatment In Chronic pain).

**Design:**

A randomized comparative-effectiveness study of antidepressants, fear-avoidance rehabilitation, or their combination in 300 patients with CLBP with high NA. In the antidepressant- or rehabilitation-only arms, SYNNAPTIC includes an adaptive design of re-randomization after 4 months for nonresponders.

**Setting:**

A multisite trial conducted in routine pain clinical treatment settings: pain clinics and physical and occupational therapy treatment centers.

**Methods:**

Inclusion criteria include CLBP with elevated depression and anxiety symptoms. Antidepressant and rehabilitation treatments follow validated and effective protocols for musculoskeletal pain in patients with high NA. Power and sample size are based on superior outcomes of combination therapy with these same treatments in a 71-subject 4-arm pilot randomized controlled trial.

**Conclusions:**

SYNNAPTIC addresses the lack of evidence-based protocols for the treatment of the vulnerable subgroup of patients with CLBP and high NA. We hypothesize that combination therapy of antidepressants plus fear-avoidance rehabilitation will be more effective than each treatment alone.

**Trial registration:**

ClinicalTrials.gov ID: NCT04747314.

## Introduction

Chronic low back pain (CLBP) affects approximately 50 million adults in the United States^1^ and is the leading cause of disability worldwide.^2^ At least 20% of patients with CLBP in primary care practices in the United States also live with major depression or an anxiety disorder.^3^^,^^4^ In patients with chronic pain, anxiety and depression symptoms commonly co-occur and are frequently accompanied by pain-specific psychological symptoms (eg, high pain catastrophizing), with correlations among these constructs ranging from 0.6 to 0.70.^5^^,^^6^ In other words, the majority of patients with CLBP and negative psychological symptoms present with broad disturbances of mental health rather than just being restricted to one domain (such as only high depression or anxiety symptoms), and negative affective disorders afflict 10–20 million patients with CLBP in the United States.^7^^–^^9^ This combination of negative emotions, thoughts, and behaviors is termed *negative affect* (NA).^6^ Patients with CLBP and high NA tend to experience greater pain and worse function and have a poorer response to pain treatments than do those with low NA.^10^^–^^12^ Patients with CLBP and high NA also have a 2× to 4× greater rate of prescription opioid misuse.^13^ “Negative affect” as a treatment target is supported by the National Institutes of Health Research Domain Criteria initiative (RDoC), which identifies “Negative Affect” (ie, the Negative Valence domain) as a needed priority for trans-diagnostic treatment interventions.^14^^–^^16^

There are numerous treatments with proven efficacy to improve both pain and NA in patients with CLBP and high NA, such as antidepressants (ADs), fear-avoidance rehabilitation, and cognitive behavioral therapy.^17^^–^^20^ Even though efficacy has been established, there is no understanding of which treatment(s), combinations, and sequences of care are most effective. The 2019 Department of Health and Human Services report, *Pain Management Best Practices Interagency Task Force Report: Updates, Gaps, Inconsistencies, and Recommendations*, highlighted the need for (1) evidence-based multimodal treatment in chronic pain and (2) additional comparative-effectiveness research to determine best practices.^21^ Therefore, we have undertaken the multisite SYNNAPTIC (SYNergizing Negative Affect & Pain Treatment In Chronic pain) randomized comparative-effectiveness trial, funded by the US National Institutes of Health (NIH) Helping to End Addiction Long-term (HEAL) initiative and the Back Pain Consortium (BACPAC) Research Program, described elsewhere in this special issue of *Pain Medicine*. In the present article, we share an overview of the clinical trial design and the supporting pilot study data. The fully detailed clinical trial protocol is available at www.ClinicalTrials.gov (ID: NCT04747314).

## Trial considerations

As noted, the evaluation of effective treatments in the subgroup of patients with CLBP with high NA is a central gap in the literature, and guidance for clinicians is lacking. Overall, although the efficacy of ADs or fear-avoidance / fear-of-movement therapy has been established in controlled trials, these treatments have not been directly compared in the high-NA subgroup of individuals with CLBP.^18^^,^^19^ This body of literature suggests multiple mechanisms related to improvement, such as direct effects on nociceptive pathways, neuromuscular stabilization, and improvements in NA leading to improvements in pain.^17^ These findings suggest that treating pain and NA together is the optimal approach to address the bidirectionality of interactions between pain and NA, although definitive evidence is lacking.

We chose to compare AD treatment and fear-avoidance / fear-of-movement rehabilitation because neither requires additional mental health services outside of what is commonly available in primary care to deliver the treatments, should they be effective (ie, scalability). We describe the fear-avoidance rehabilitation in SYNNAPTIC as *application*-*enhanced* fear-avoidance rehabilitation (AEFAR) because, in addition to the classical approach of graded exercise and exposure, we include a pain self-management/education tool delivered by a mobile application, which reinforces the treatment sessions (see pilot data in the section “Pilot study results from a pre-SYNNAPTIC 4-arm randomized trial”). Studies have shown that fear-avoidance rehabilitation is effective in part through targeting multiple domains of NA, such as kinesiophobia, pain catastrophizing, and anxiety.^22^^,^^23^ Subjects are unlikely to have had AEFAR, and the AD protocol provides several options for ADs that the participants have not tried, which addresses the potential confounder of prior treatment exposure.

To further improve the generalizability and scalability of our findings, we modified an AD treatment regimen from Kroenke et al. that was proved effective in primary care in the treatment of patients with musculoskeletal pain and depression (the Stepped Care for Affective Disorders and Musculoskeletal Pain [SCAMP] trial).^18^ This algorithm potentially involves the use of any AD, because the first step is to optimize the dose of a current AD the participant might already be taking at enrollment, if applicable. The modification to the SCAMP protocol is to use duloxetine or venlafaxine (serotonin–norepinephrine reuptake inhibitors) as a second step because of their efficacy in treating pain.^24^^–^^26^ This modified approach will make our findings immediately applicable to primary care physicians and spine care specialists. Most clinically impactful comparative-effectiveness studies in chronic pain include explanatory and pragmatic trial design elements,^27^ and we are taking this approach to achieve a balanced design. In addition, we followed the guidelines of the IMMPACT group (Initiative on Methods, Measurements, and Pain Assessment in Clinical Trials) for proof-of-concept studies.^28^

## Trial design

**Figure 1.**
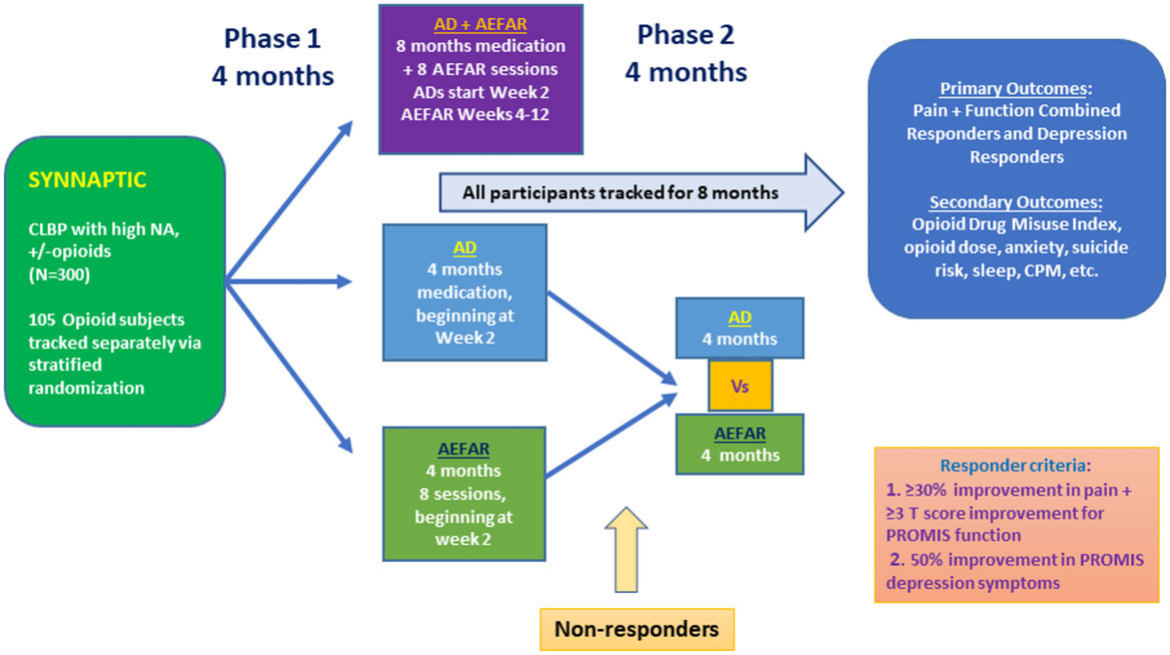
SYNNAPTIC trial schema. AD = antidepressant; AEFAR = application-enhanced fear-avoidance rehabilitation; CPM = conditioned pain modulation; CLBP = chronic low back pain; NA = negative affect.

A 3-arm design is ideal to evaluate the comparative effectiveness of combination therapy in pain trials.^29^^,^^30^ We will test improved stepped care with ADs vs AEFAR vs AD+AEFAR after 4 months of treatment (phase 1), which will determine the separate and additive effects of the interventions (see Figure 1). Phase 2 is treatment continuation in AD+AEFAR to assess treatment durability and the adaptive design element of re-randomization of nonresponders to AD or AEFAR alone in phase 1 to enrich the pool of responders for secondary analyses. Our primary hypothesis is that AD+AEFAR will show the most improvement in pain, function, and depression at the end of phase 1 as compared with AD or AEFAR alone. ADs are prescribed by nurse practitioners with oversight from the coinvestigators, the rehabilitation is delivered by physical or occupational therapists practicing in routine settings, and the research procedures are embedded directly in clinical treatment settings, all of which are pragmatic components of the trial design.

The primary outcome and endpoint in phase 1 is a composite measure of responder status that categorizes responders on the basis of improvements in pain, function, and depression. Responder status is based on Patient-Reported Outcomes Measurement Information System (PROMIS) measures at 4 months vs baseline, and responders are categorized as (1) combined responders who have both pain and function improvements and/or (2) depression responders. A participant could be a combined responder, a depression responder, both, or neither. We use accepted benchmarks for clinically meaningful improvement in CLBP and NA that have been validated as outcomes in chronic pain studies.^31–36^ These benchmarks are (1) an improvement of at least 30% in average daily pain on the PROMIS average pain intensity question; (2) an improvement of at least 3 points in the PROMIS T score for function (a minimal clinically important difference); and (3) an improvement of at least 50% in depression symptoms, which is a PROMIS T-score improvement of at least 5 points.

**Figure 2.**
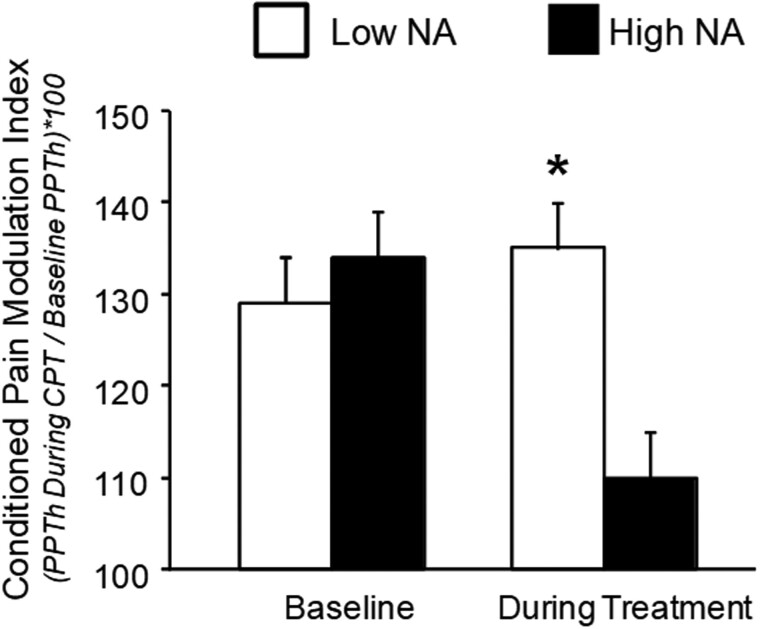
Conditioned pain modulation in low- and high-NA groups. Data presented as group means ± SEM.^39^ *Groups differ at *P* < 0.05 on nonparametric Mann-Whitney *U* tests. CPT = conditioned pain testing; NA = negative affect; PPTh = pressure pain threshold testing.

Secondary outcomes in phase 1 focus on the clinical and mechanistic links among pain, NA, and prescription opioids. As patients with CLBP and high NA are more frequently prescribed opioids at higher doses and are more likely to misuse them, opioid dose and misuse are important secondary outcomes.^13^ These are expected to lessen as pain and NA improve, and a secondary hypothesis is that AD+AEFAR will be the most effective in preventing opioid misuse and will be associated with greater reductions in daily opioid dose vs AD or AEFAR alone. We have shown that patients with CLBP and high NA have impaired conditioned pain modulation (CPM), which is a quantitative sensory measure of endogenous analgesic systems that serve to dampen the perception of pain.^37^ Patients with lower CPM are more sensitive to pain and have more clinical pain.^38^ We have also shown that in the course of opioid treatment, CPM drops precipitously in patients with CLBP and high NA (Figure 2).^39^ As such, another secondary hypothesis is that improvements in CPM will precede improvements in pain and mediate treatment responses in those prescribed opioids.

Patients already prescribed opioids at enrollment will be sub-stratified during randomization so that we can more adequately test the secondary hypotheses that targeting high NA is effective to reduce opioid misuse and improve CPM. The final list of secondary outcomes includes changes in domains within the BACPAC minimum data set, neuropathic symptoms (measured with PainDetect), and opioid misuse (Table S1).

Another secondary aim of SYNNAPTIC is to better characterize responders to each of the 3 treatments (AD, AEFAR, or AD+AEFAR). We hypothesize that participants with relatively elevated baseline scores on a measure of depressive symptoms will respond best to ADs and that participants with relatively lower levels of baseline physical functioning will respond best to AEFAR*.* We need as large a pool as possible of responders to each treatment to better identify such predictors of responses. Therefore, phase 2 will include the adaptive component for participants in the AD- or AEFAR-only arms in phase 1 not meeting the benchmarks for clinically meaningful improvement, who will then be re-randomized to continue or switch to the other treatment for 4 months (phase 2). We track outcomes for 8 months in all treatment arms to assess intervention durability (Figure 1).

To minimize burden on the participants, we will assess outcomes with weekly ratings through emailed and texted RedCap surveys and monthly in-person visits (synchronized with AEFAR sessions when possible). In addition, the AD adjustment visits occur every 2 weeks via phone calls, and the 8 AEFAR sessions can be completed by 4 in-person and 4 telemedicine-delivered sessions if requested (Tables S1 and S2).

### Inclusion/exclusion criteria

From our previous studies in CLBP,^13^^,^^40–43^ we devised broad inclusion criteria that will improve the generalizability of our results and minimize confounders. These criteria meet the recommended NIH standards for CLBP research.^44^

#### Inclusion

1. Ages 18–75 years. The risk of cognitive impairment significantly increases above age 75 years.
2. Pain characteristics: (A) Duration >6 months, as this is unlikely to spontaneously improve^45^; (B) average pain score within the past week of >3/10; and (C) low back pain is the primary pain site.
3. CLBP meeting Quebec Task Force Classification System categories I–III (from axial pain only to pain radiating beyond the knee without neurological signs).^46^^,^^47^ Constant radicular pain associated with sensory loss is highly treatment resistant without surgery^48^ (assessed by the NIH Research Standards for CLBP).^44^
4. A prior lumbar spine X-ray to screen out red flags such as tumor, infection, or fracture.^49^ Most subjects will have had one, as it is the standard of care to obtain an X-ray to exclude unusual causes of treatment-refractory pain of longer than 6 months’ duration.^49^
5. High NA at the first visit (with symptoms for at least 1 month), defined as >85th percentile on both the depression and anxiety modules of PROMIS. These scores correlate highly with having a comorbid major depression or generalized anxiety disorder.^50^^,^^51^
6. Receiving medical care from the University of Pittsburgh Medical Center, Brigham and Women’s Hospital, or Mayo Clinic, Rochester, so that monthly visits, usual care, other treatments, and health care utilization can be tracked within the electronic medical record.
7. Access to the Internet and a smartphone. Nationwide reports show that 85%–94% of low-income families have Internet access at home.^52^^,^^53^ Data indicate that on average, 77% of all Americans across all socioeconomic, racial, age, and demographic groups have a smartphone.^54^
8. For the opioid inclusion group who have sub-stratified randomization, those subjects must be prescribed opioids currently on a monthly basis for at least 3 consecutive months. This criterion will include those prescribed opioids daily, as well as those taking opioids intermittently during the week.
9. Ability to understand English and comprehend the questionnaires.

#### Exclusion

1. Back surgery within the prior 6 months, as this is within the window of normal healing.
2. Psychiatric: (A) Acute suicidality, (B) history of bipolar disorder, or (C) history of psychosis. These conditions would require treatment other than the AD medication provided in the study. They will be assessed at study entry and through a review of history in the electronic medical record.
3. Pregnancy or intent to become pregnant during study, as which AD to use in pregnancy is controversial. Child-bearing females will have to provide assurances that they use contraception if sexually active.
4. Intent to add new pain or psychiatric treatments during phase 1 (except for what is part of the study).
5. Intent to have back surgery during phase 1.

### Recruitment

We will use multiple pathways for recruitment depending on the preferred methods at each clinical site. These include patient volunteer registries, clinic advertising, electronic bulletin boards, and best practice alerts in the electronic medical records.

### Visit schedule, randomization, and study procedures

See Table S1 for more information.

#### Telephone screening and study visits 1 and 2 (weeks 0–2)

At their phone call, all interested volunteers will be asked to complete the Patient Health Questionnaire 4 (PHQ-4), which is a 4-item brief measure of depression and anxiety symptoms and is highly accurate for predicting a depression or anxiety disorder.^55^^,^^56^ Those with elevated depression and anxiety symptoms above the 85th percentile and meeting the other eligibility criteria will be scheduled for visit 1. At visit 1, we will obtain written informed consent, the subjects complete the baseline assessments, and we reconfirm the eligibility criteria. The study advanced practice providers or coinvestigators will conduct the clinical evaluation, which includes medical and psychiatric histories, review of history in the electronic medical record, and an exam if necessary. We use the Mini International Neuropsychiatric Interview (MINI) structured interview module for substance use disorders^57^ to rule out a current substance use disorder (in conjunction with obtaining a urine drug test in the subjects taking opioids). We used the same battery in our AD+AEFAR pilot and a previous study,^13^ and it took an average of 45 minutes to complete. In the rare instance of subjects presenting with severe psychiatric symptoms, such as psychosis or active suicidality, at any time point in the study, the study physicians will undertake the appropriate safety steps to safeguard the subject, who will be withdrawn from further study and referred for appropriate treatment.

At visit 2 in week 2, urine drug test results are reviewed to confirm study inclusion (if needed), surveys are completed, and opioid prescribing is coordinated with the current opioid prescriber, as needed. AD or AEFAR treatment begins, and medication is free. We track whether any new pain or psychiatric treatments are started during the study with a weekly self-completed log.

#### Baseline pain, function, and NA observation period

The PROMIS-29 scores at visits 1 and 2 (1 week apart) are averaged to establish the baseline levels of pain, function, and NA, as recommended.^58^

#### Randomization and re-randomization in nonresponders

Before visit 2, the investigational drug pharmacy at each site will randomize subjects in blocks of 6 to an equal allocation schedule: (A) AD only, (B) AEFAR only, or (C) AD+AEFAR. The randomization will be sub-stratified by opioid status. Responders at 4 months in any arm will continue to be tracked for 8 months. *Nonresponders* are those not meeting the combined pain+ function metric and the depression response metric (at least 50% improvement in symptoms). They will be re-randomized in equal blocks of 4 to continue treatment or switch to the other treatment for 4 months (Figure 1).

#### Subsequent visits

Subjects in the AEFAR and AD+AEFAR arms have 10 visits over 4 months (study visits at weeks 1 and 2; 8 AEFAR sessions; plus study visits at months 2 and 4 synchronized with AEFAR visits). Because the key outcomes are collected remotely via RedCap, only the first- and second-week study visits are required in person in any of the arms. For the AD-only arm, there are 4 visits over 4 months for the non-opioid group, and there are 5 visits for opioid subjects because of the monthly weaning visits.

#### Sequential care in the AD+AEFAR arm

ADs start at week 2, and AEFAR starts in week 4. As suggested by our preliminary data, when ADs are started 2 weeks before AEFAR, it is more likely that early improvements in pain and NA could translate into greater benefit from AEFAR to further improve pain, function, and NA. Responders and nonresponders to AD+AEFAR continue ADs for 8 months total. From months 5–8, we will do an abbreviated assessment every 2 weeks via an email and text message with a Web link to the surveys.

#### Conditioned pain modulation

CPM procedures require a conditioning stimulus to induce endogenous analgesic systems and alter pain perception and a test stimulus to evaluate the endogenous analgesic response to the conditioning stimulus. We will use the same CPM testing procedures as in our previous study in patients with CLBP and high NA who were prescribed opioids.^39^ This is done at visit 2 and at months 2 and 4. The re-randomized nonresponders will have additional testing at months 6 and 8. Immersion of one hand into a circulating cold water bath (4°C–10°C; NESLAB Digital One RTE 7, Thermo Scientific, Newington, NH, United States, or similar) will serve as the conditioning stimulus, and pressure pain threshold at the contralateral trapezius will serve as the test stimulus.

Pressure pain threshold is assessed with a hand-held algometer with a 1-cm^2^ rubber probe (FPK20 or FPX25, Wagner Instruments, Greenwich, CT, United States) over the upper trapezius muscle contralateral to the participant’s dominant hand. Pressure is manually increased at a rate of rise of 0.5 kgf/cm^2^/s (10 kgf/cm^2^ maximum, metronome guided) until participants first report that the pressure sensation becomes painful. Pressure intensity (in kgf/cm^2^) read from the algometer at that time is considered the pressure pain threshold. Measurements are conducted 3×/site with 60-second rest intervals between each pressure application. Probe placement is varied slightly from trial to trial to prevent sensitization from repeated testing of the same site. Mean pressure pain threshold is used for analysis.

Conditioning stimulation will begin by immersing the hand to a level 10 cm above the wrist into the water bath. The hand will be immersed for a total of 30–60 seconds of hand immersion, and trapezius pressure pain threshold will re-measured 1–2 times while the hand is still immersed in the cold water. CPM magnitude will be calculated as the difference in mean pressure pain threshold measured before and during the conditioning stimulus, with increases in pressure pain threshold during conditioning interpreted as evidence of efficient endogenous pain inhibition.

#### Assessment of treatment adherence, credibility, and tolerability

We will track AEFAR session attendance, AD adherence, use of self-management techniques, and any new pain or psychiatric treatments. We will also use audits of EPIC records to track these factors and the study withdrawal form in dropouts. Subjects will rate the usefulness of the interventions in all study arms at study visits with the Treatment Helpfulness Questionnaire (THQ).^59^

### Treatment procedures

#### Antidepressants

There are published protocol details for SCAMP, which we have modified for SYNNAPTIC.^18^^,^^60^^,^^61^ Advanced practice providers prescribe ADs with oversight from study medical doctors. We use the Antidepressant Treatment History Form^62^ to guide AD selection, and AD prescribing begins at visit 2 after a 1-week baseline pain and mood observation period (see Table S1). Medication is dispensed from the investigational drug pharmacy. SCAMP optimized AD therapy and was not designed to sort out differences among individual agents. For instance, if a subject is on an AD at study entry and the dose should go higher, it is then increased (see Table 1). If subjects are already on a suitable dose, then they are weaned off of the AD over 1–2 weeks, and a new one is started according to the flexible algorithm. Although unlikely, it is possible that a subject could be on an AD at a suitable dose that improved pain but not depression or anxiety, as ADs can improve pain independent of mood effects.^63^^,^^64^ In this scenario, the study prescriber will proceed to step 4, augmentation of AD. SYNNAPTIC has no strict hierarchical algorithm because only one-third of patients started on any particular AD achieve remission,^65^ and there is no strong evidence that one AD is superior to another.^66^^,^^67^ This flexible prescribing approach is standard in AD trials, such as STAR*D, and mirrors clinical practice.^65^^,^^67^ Serotonin and norepinephrine reuptake inhibitors (eg, venlafaxine or duloxetine) are step 2 because of their analgesic properties.^68^^,^^69^ Because of potential cytochrome p450 interactions with opioids,^70^ SYNNAPTIC will use only sertraline and citalopram as selective serotonin reuptake inhibitors in step 3. Step 4 treatment is bupropion or mirtazapine (added to or as a single AD) or aripiprazole for augmentation, depending on symptoms, such as fatigue, weight gain, or insomnia.

**Table 1:** SYNNAPTIC AD medication steps.

**Step 1:** If currently on an AD, optimize the dose if possible
**Step 2:** Venlafaxine or duloxetine
**Step 3:** Escitalopram or sertraline if the patient had already been on an SNRI or if it failed
**Step 4:** If needed, depending on symptoms, bupropion, mirtazapine, or apiprazole as augmentation or stand-alone medications

Abbreviations: AD = antidepressant; SNRI = serotonin and norepinephrine reuptake inhibitors.

The prescriber maximizes the AD dose as tolerated up to a clinical endpoint of at least 50% improvement in depression or anxiety symptoms and at least 30% improvement in pain. There are 4 telephone contacts (one every 2 weeks) up until week 16, in which the study prescriber discusses the medication and makes adjustments. The phone call is structured with standardized assessment measures (Table S1) to discuss symptoms, side effects, and dose adjustments, and it lasts at least 10 minutes. There are no strict guidelines for the AD doses prescribed. Side effects are monitored and responded to at each contact, including suicidality.^71^ Suicidality is also assessed at baseline and at 8 and 16 weeks in phase 1 via the Columbia Suicide Severity Rating Scale (CSSRS).^72^ AD responders at 4 months continue medication for 8 months total. At the end of the study, subjects are given the option of continuing ADs in discussion with their pain medicine medical doctor or their primary care provider. Nonresponders in the AD-only treatment arm who are re-randomized at 4 months will be given the option of continuing the AD for 4 more months or being weaned off in discussion with the study prescriber.

#### Application-enhanced fear-avoidance rehabilitation

AEFAR includes graded exposure, activity, exercise, pain education, motivational interviewing techniques, and a smartphone app, which together drive behavioral change. Treatment is delivered by trained physical or occupational therapists. AEFAR starts at week 2 or 4 (depending on the study arm), is completed in 8–12 weeks, and includes: (1) A physical therapy examination (start and at end of 8 treatments), (2) Fear of Daily Activities Questionnaire (FDAQ) (baseline and monthly—tracked as an outcome),^73^ and (3) eight 45-minute physical therapy / occupational therapy sessions 1–2 times per week. The degree of graded exposure to functional activities is determined by using the FDAQ, which is a list of 10 activities patients with CLBP commonly report being fearful of performing (Table S2). The therapist identifies highly rated items, which becomes the focus of the therapy. AEFAR also includes verbal and written educational material on CLBP and fear of movement, which is important to reduce fear-avoidance behaviors.^74^ As per the validated protocol, an individualized home program is developed. A portion of the treatment visits are audited by the site principal investigators to assess treatment fidelity.

#### Opioid weaning and measuring misuse

Subjects who were already prescribed opioids at enrollment are given the option to voluntarily reduce their opioids if desired, supervised by a pain specialist, at an individually tailored rate of 10%–25% per month. As in clinical practice, weaning is done individually, and participants are seen once per month if they are being weaned from chronic opioid therapy.^75^ We anticipate that if the patients are benefiting from treatment, they will be more willing to wean and will be able to do so without increases in pain. As in our previous low- vs high-NA opioid treatment study,^13^ separate study physicians who are blinded to treatment group assignments will assume the opioid prescribing at the current dose, and thus prescribing practices and adherence monitoring will be standard across the 3 arms. All subjects sign an opioid therapy agreement, and at baseline and months 2 and 4, we will collect the Drug Misuse Index (DMI), which we developed, validated, and used in several prior studies.^76–79^ Maintaining the blind could be a source of bias, and we will ask the physicians to which treatment group they think each subject was assigned. To better categorize the prevention of misuse, we will classify it as: (1) Reversed (misuse at baseline but none at 3 or 6 months), (2) avoided (ie, no misuse at any time point), (3) unchanged (misuse at baseline and at follow-up points), or (4) present (no misuse at baseline but positive misuse at 3 or 6 months).

### Pilot study results from a pre-SYNNAPTIC 4-arm randomized trial

To investigate feasibility of the protocol and to generate data for sample size calculations, we used the same inclusion/exclusion criteria to conduct a randomized trial in 71 subjects with CLBP and high NA (on or off opioids). We followed all approved institutional review board procedures of the University of Pittsburgh, including obtaining written informed consent from each participant. The HEAL/BACPAC program emphasizes the need for robust trial planning and pilot data, which are considered vital parts of protocol development. Subjects were randomized, in a 1:1:1:2 ratio, to ADs (AD; via the same treatment algorithm), fear-avoidance–based physical therapy (FABPT), pain education (which served as an active control), or the combination of AD+FABPT. FABPT did not include the pain education mobile app incorporated into AEFAR, but otherwise the content and delivery of the treatment sessions were the same. The pain education control condition used the content from Dr. Michael Hooten’s book, *The Mayo Clinic Guide to Pain Relief,*^80^ which is a pain self-help book that is highly rated by patients.^81^ It focuses on CLBP, and the topics are (1) Chronic Pain as an Illness to be Managed, (2) How Chronic Pain Affects You, (3) Treating Chronic Pain, (4) Healthy Lifestyles, and (5) Setting Goals. It was delivered by a trained research assistant in 8 weekly sessions over 2 months. We used the same responder benchmarks, treatment procedures, outcomes tracking, and primary outcomes as in SYNNAPTIC.

**Figure 3.**
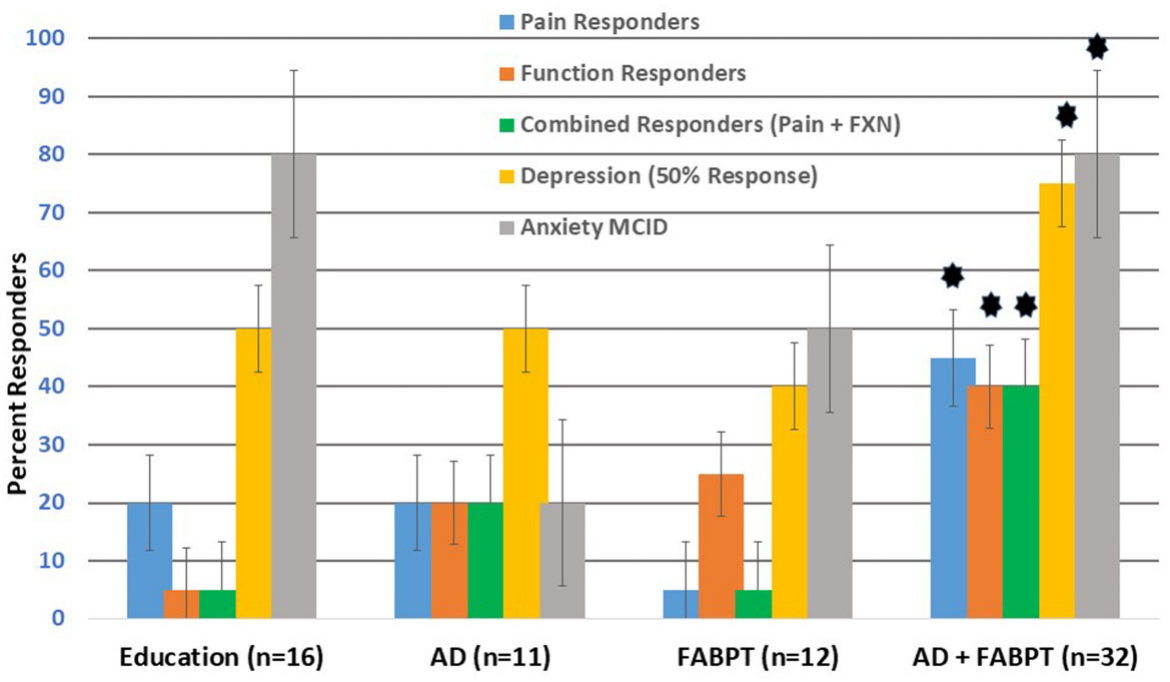
Pilot study results in 71 subjects with CLBP and high NA. Data presented as proportion of responders ± SE. *AD+FABPT group differs at *P* < 0.05 on nonparametric Fisher exact tests vs the AD, FABPT, and education-only treatment groups. AD = antidepressants; FABPT = fear-avoidance–based physical therapy; FXN = function; MCID = minimum clinically important difference.

There was an average 80% study completion rate in the 4 arms. Numbers of enrolled subjects were as follows: pain education, n = 16; AD, n = 11; FABPT, n = 12; and the combination group, n = 32. Figure 3 shows that AD+FABPT had a 40% responder rate for pain ± function and that AD+FABPT had a 75% depression responder rate (*P* values <.05 for the Fisher exact tests vs the other groups). The combination of AD+FABPT was the most effective across the key domains (pain, function, anxiety, and depression) in patients with CLBP and high NA, and these data formed the basis for our sample size calculations.

### Statistical methods and power

The primary comparisons of interest in SYNNAPTIC (ie, the primary outcomes) are the proportion of pain + function (combined) responders and depression responders between study treatment arms at the end of 4 months of phase 1 (nonopioid + opioid subjects together). None of the phase 2 data will be used for the primary outcomes analysis. We hypothesize that the proportions of combined and depression responders will be significantly greater in the AD+AEFAR arm than in the other arms. Therefore, 2 primary pairwise comparisons will be made simultaneously: (1) AD+AEFAR vs AD alone and (2) AD+AEFAR vs AEFAR alone. In our pilot study, the combined responder rate was 40% in the AD+FABPT arm, 20% in the AD arm, and 25% in the FABPT arm. We will assume that in the proposed study, the rate of combined responders will be 20% in the AD-or AEFAR-only groups and 50% in the AD+AEFAR group. With equal sample size allocation among the 3 arms and with a 2-sided significance level of 0.025 for each comparison after Bonferroni adjustment, a sample size of 240 study completers (80 per arm) will provide about 95% power to detect a minimum difference of 0.30 from the baseline value of 0.20 in response rate for each hypothesis testing. After adjustment for a dropout rate of 20% at 4 months, the final sample size at enrollment will be about 300, ie, 100 per each arm. Subjects with missing data will be included in the data analysis after an intention-to-treat analysis, provided that they had treatment exposure. We will also perform an as-treated analysis and analyze the secondary outcomes similarly.

For the secondary opioid outcomes in phase 1, we will compare the proportions of opioid misuse and dose changes between subjects by using simple descriptive statistics. Combining phase 1 and 2 data, we will use mediation analysis to investigate whether changes in CPM mediate changes in pain in those who were prescribed opioids, and we will perform a cross-lagged panel analysis to test whether changes in CPM precede changes in pain.

In phase 2, the re-randomization of nonresponders to AD or AEFAR is an adaptive element to maximize an enriched pool of responders to any treatment in both phases. We anticipate in phase 1 to generate 50 AD+AEFAR responders, 20 AD responders, and 20 AEFAR responders, based on 100 enrolled subjects per arm. In phase 2, we anticipate 12 more AD and 12 more AEFAR responders, and thus, we anticipate having a total of 50 AD+AEFAR, 32 AD, and 32 AEFAR responder patients for analysis, vs 186 nonresponders (composed of study dropouts and actual nonresponders to treatment). We will use logistic regression to build a prediction model including up to a maximum of 10 significant predictors via a variable selection method such as LASSO. The prediction error will be assessed and presented by use of the Brier scores.^82^

#### Missing data and treatment adherence

We will track subject attrition by treatment arm. We will inspect the Study Withdrawal Case Report Forms at least monthly to monitor and limit the amount and impact of missing data and to understand whether data is missing not at random. We will pay attention to dropout rates in substrata of subjects, such as those with higher psychiatric or pain symptoms and those on opioids. Analyses for handling missing data require assumptions about the type of “missingness,” and we will apply imputation methods accordingly.

Missing data and treatment adherence might be particularly important issues during the COVID pandemic and even after it resolves. We anticipate that (1) recruitment to participate in the study could be challenging because there are some in-person required visits; (2) we will have to deliver the treatments as remotely as possible; (3) we will need to be flexible in application of the protocol so as to maximize study retention, treatment adherence, and data collection; and (4) staffing shortages that affect health care and medical schools could also impact recruitment, retention, and data acquisition quality.

## Conclusions

SYNNAPTIC addresses the lack of evidence-based guidelines for the optimal treatment approach in the prevalent subgroup of patients with CLBP and high NA. Our anticipated findings will provide high-quality evidence that can be incorporated into treatment guidelines. If successful, SYNNAPTIC will provide an effective pathway for treating psychiatric comorbidity in CLBP in routine medical settings that does not require additional mental health services. This suggests that SYNNAPTIC can be scaled up effectively and rapidly at the community level.

## Supplementary Material

pnad006_Supplementary_Data
