# Supplementary material for: Back Pain Consortium (BACPAC): Protocol and Pilot Study Results for a Randomized Comparative-Effectiveness Trial of Antidepressants, Fear Avoidance Rehabilitation, or the Combination for Chronic Low Back Pain and Comorbid High Negative Affect
Source: Pain Med. 2023 Jan 30;24(Suppl 1):S105–14. doi: 10.1093/pm/pnad006 (PMC10403304; doi:10.1093/pm/pnad006)
Supplement: pnad006_Supplementary_Data [file pnad006_supplementary_data.zip › pnad006_Supplementary_Data/supplemental table 1.docx]

**Supplementary Table 1: SYNNAPTIC Study Schedule of Questionnaires/Key Study Steps**

| **Schedule of Events for Research Subjects** | | | | | | | | | |  |  |  |  |  |  |
| --- | --- | --- | --- | --- | --- | --- | --- | --- | --- | --- | --- | --- | --- | --- | --- |
| **Procedures and Forms** | Phone Screening | Baseline | Weekly Surveys | Antidepressant Treatment (Wk 2 - Month 8) | | | | | |  |  |  |  |  |  |
|  |  |  |  | AEFAR (AD+AEFAR arm Wk 2-12) | | | |  |  |  |  |  |  |  |  |
|  |  |  |  |  | AEFAR (AEFAR only arm Wk 4-12) | | | |  |  |  |  |  |  |  |
| **Week** | 0 | 1 | 1-16 weekly, 17-32 bi-weekly | 2 | 4 | 8 | 10 | 12 | 16 |  |  |  |  |  |  |
| **Month** | 0 | 1 | 1 - 8 | 1 | 2 | | 3 | | 4 |  |  |  |  |  |  |
| **Study Visit** | -- | 1 | -- | 2 | -- | 3 | -- | -- | 4 |  |  |  |  |  |  |
| **Study Events** |  | Randomize |  |  |  |  |  |  | Primary endpoint |  |  |  |  |  |  |
| **Questionnaires/Measures** |  | | | | | | | | |  |  |  |  |  |  |
| Consent Form |  | X |  |  |  |  |  |  |  |  |  |  |  |  |  |
| Demographics (BACPAC required) | T | X |  |  |  |  |  |  |  |  |  |  |  |  |  |
| BACPAC minimum dataset |  | X |  |  |  | X |  |  | X |  |  |  |  |  |  |
| PHQ-4 | T |  |  |  |  |  |  |  | X |  |  |  |  |  |  |
| Drug Misuse Index |  | X |  |  |  | X |  |  | X |  |  |  |  |  |  |
| Urine Pregnancy (ADs arms only) |  | X |  |  |  |  |  |  |  |  |  |  |  |  |  |
| Urine Drug Toxicology (in those on opioids only) |  | X |  |  |  | X |  |  | X |  |  |  |  |  |  |
| General Health / Medical History (with drug allergies) | T | X |  |  |  |  |  |  |  |  |  |  |  |  |  |
| Antidepressant Treatment History Form (ATHF) |  | X |  |  |  |  |  |  |  |  |  |  |  |  |  |
| TAPs Tool 1 & 2 (for substance use history) |  | X |  |  |  | X |  |  | X |  |  |  |  |  |  |
| Current Opioid Misuse Measure (COMM) |  | X |  |  |  | X |  |  | X |  |  |  |  |  |  |
| Addiction Behaviors Checklist (ABC) |  | X |  |  |  | X |  |  | X |  |  |  |  |  |  |
| Self-Administered Co-Morbidity Questionnaire |  | X |  |  |  |  |  |  |  |  |  |  |  |  |  |
| Medication Log |  | X | I | X |  | X |  |  | X |  |  |  |  |  |  |
| Opioid Side Effect Checklist |  | X |  | X |  | X |  | X | X |  |  |  |  |  |  |
| Expectations/Pre-Treatment Assessment Form |  |  |  | X |  | X |  |  | X |  |  |  |  |  |  |
| Treatment Helpfulness Questionnaire |  |  |  | X |  | X |  |  | X |  |  |  |  |  |  |
| Patient Global Impression of Change (PGIC) |  |  |  |  |  | X |  |  | X |  |  |  |  |  |  |
| **Pain/Function Questionnaires** |  | | | | | | | | |  |  |  |  |  |  |
| PROMIS-29 (Pain Intensity, Pain Interference, Physical Function) |  | X | I | X |  | X |  |  | X |  |  |  |  |  |  |
| Pain Self-Management Log | T | X | I | X |  | X |  |  | X |  |  |  |  |  |  |
| Activity Levels (via Fitness Tracker, optional) |  | X | I |  |  |  |  |  |  |  |  |  |  |  |  |
| PROMIS Pain Behavior |  | X |  | X |  | X |  |  | X |  |  |  |  |  |  |
| Widespread Pain Index (WPI) + Symptom Severity (includes body map) |  | X |  | X |  | X |  |  | X |  |  |  |  |  |  |
| PainDETECT |  | X |  | X |  | X |  |  | X |  |  |  |  |  |  |
| PEG Pain Interference |  | X |  | X |  | X |  |  | X |  |  |  |  |  |  |
| **Psychological/Behavioral Questionnaires** |  | | | | | | | | |  |  |  |  |  |  |
| Fear of Daily Activities Questionnaire (FDAQ, at every AEFAR session only, not the study visits) |  |  |  | X | X | X | X | X |  |  |  |  |  |  |  |
| PHQ-9 (used for titration of AD meds) |  |  | I (every other week) | X |  |  |  |  |  |  |  |  |  |  |  |
| PROMIS-29 (Depression, Anxiety, Sleep, social role) |  | X | I | X |  | X |  |  | X |  |  |  |  |  |  |
| PROMIS-2 Item Cognitive Screener questions |  | X |  |  |  |  |  |  |  |  |  |  |  |  |  |
| Columbia-Suicide Severity Rating Scale (CSSRS)  *****Assessed by a nurse practitioner |  |  |  | X |  | X |  |  | X |  |  |  |  |  |  |
| Fear Avoidance Beliefs Questionnaire—physical activity items (FABQ-PA) |  | X |  | X |  | X |  |  | X |  |  |  |  |  |  |
| Pain Catastrophizing Scale (6-items PCS) |  | X |  | X |  | X |  |  | X |  |  |  |  |  |  |
| Opioid Craving Questions |  | X | I | X |  | X |  |  | X |  |  |  |  |  |  |
| -AD Visits |  |  |  |  |  |  |  |  |  |  |  |  |  |  |  |
| Start Rx given for AD |  |  |  | X |  |  |  |  |  |  |  |  |  |  |  |
| AD Side Effects Checklist |  |  | I (every other week) |  |  |  |  |  |  |  |  |  |  |  |  |
| AD side effect burden (FIBSER) |  |  | I (every other week) |  |  |  |  |  |  |  |  |  |  |  |  |
| -AEFAR Visits |  |  |  |  |  |  |  |  |  |  |  |  |  |  |  |
| Use Vivify App (at end of AEFAR treatment sessions) |  |  |  |  |  |  |  | X |  |  |  |  |  |  |  |
|  |  | | | | | | | | |  |  |  |  | X |  |
| **Remuneration (parking, bus, subway costs covered for study visits). Treatments are free** |  | 25 |  | 25 |  | 25 |  |  | 25 |  |  |  |  |  |  |
|  |  |  |  |  |  |  |  |  |  |  |  |  |  |  |  |

**T=Telephone; I=Internet/Web Based; X=In-Person; Yellow=Opioid users only; Green=EFAR or EFAR+AD users only; Blue=Antidepressant users only**
